# Optimizing Breast Implant Outcomes: MemoryGel Xtra Implants and Future Research Directions

**DOI:** 10.1093/asj/sjae061

**Published:** 2024-03-18

**Authors:** Shahin Benyaminpour, Moshe Shalom

We read with great interest “Clinical Results of Mentor MemoryGel Xtra Breast Implants From the GLOW Clinical Trial” by Alderman et al.^1^ The clinical trial very aptly showed the benefits of MemoryGel Xtra implants (Mentor, Irvine, CA) and the low complication rates associated with the implants. The cohort was subdivided into 4 categories: primary augmentation, revisional augmentation, primary reconstruction, and revisional reconstruction. Within these groups, different rates of complications were observed. Furthermore, the levels of satisfaction with the breasts, psychosocial well-being, sexual well-being, and physical well-being were analyzed preoperatively and postoperatively at the 1-year mark. Across the board satisfaction with breasts, as well as psychosocial and sexual well-being, were reported to be significantly elevated 1 year after the operation. Notably, there were no reported cases of malposition or displacement, infection, rupture, or wrinkling or rippling, showcasing the reliability and safety of these implants.

MemoryGel Xtra implants are seen to be more beneficial because they optimize the shell gel filling.^2^ The authors draw attention to the concept that increasing the degree of implant shell filling may reduce the incidence of clinically significant wrinkling and rippling. This concept is grounded in experiences with saline-filled implants, with which maximal filling has been associated with reduced wrinkling and rippling and a prolonged implant life span.^3^ The design of MemoryGel Xtra implants incorporates this idea, optimizing gel fill to enhance central device fullness and projection while minimizing surface wrinkling. The authors express their expectation that continued observation over the next 7 years will reveal whether these precision-filled implants indeed lead to enhanced long-term results. Shell gel filling, as stated by the authors, may have a major impact on future breast complications, and this is an area that should be further explored in future studies.

With regard to deflation rates, a study done by Eisenberg showed that females with breast implants filled above the manufacturers recommended fill volumes as opposed to those within the recommended fill volumes showed a significantly reduced deflation rate.^3^ Furthermore, in another study done by Al-Sabounchi, it was shown that filling Mentor 2600 prostheses to a level above the recommended guidelines led to increased longevity of the breast implants and as well had a significant effect on the 10-year survival rate of the implants.^2^ Future research into determining the optimal levels for shell filling of MemoryGel Xtra implants and their impact on various complications can potentially contribute to increased overall performance of breast implants and can guide the development of even more advanced and tailored devices.

Moreover, an area that might be beneficial to further explore would be the comparative outcomes between MemoryGel Xtra implants and other implants in the same class from companies like Allergan and Sientra. An in-depth investigation into overfilling techniques with the breast implants of other manufacturers in correlation with Mentor MemoryGel Xtra implants might provide further insight into the long-term incidence of wrinkling, rippling, deflation, and other consequences of breast implantation and augmentation. Acknowledging the existence and potential research interest in other breast implant technologies would present a more balanced view and enrich the discussion. Additionally, comparative studies would provide a more comprehensive understanding of breast implants in the industry.

Last, the authors should advocate for participation in national breast implant registries as a form of acquiring this much-needed data. By encouraging participation in such registries, researchers can gather valuable real-world evidence that can further inform clinical practice and improve patient outcomes.

We commend the authors on their findings. The results showed the benefits of MemoryGel Xtra implants and their low complication rates. The findings from the MemoryGel Xtra cohort analysis open avenues for future research that can deepen our understanding of breast implant technologies and their impact on patient outcomes. We believe that future studies to update the literature should look at the optimal shell gel filling levels in conjunction with postoperative outcomes. By addressing the outlined areas, researchers can contribute to the refinement of implant design, surgical techniques, and postoperative care, ultimately enhancing the safety, satisfaction, and long-term success of breast augmentation procedures.
